# Bibliometric and visualized analysis of drug resistance in ovarian cancer from 2013 to 2022

**DOI:** 10.3389/fonc.2023.1173863

**Published:** 2023-05-18

**Authors:** Jiahua Liu, Junnan Ma, Jiarong Zhang, Chengming Li, Bowen Yu, Hyok Chol Choe, Kaiyue Ding, Liu Zhang, Lin Zhang

**Affiliations:** Institute (College) of Integrative Medicine, Dalian Medical University, Dalian, China

**Keywords:** drug resistance, ovarian cancer, bibliometric, Citespace, Vosviewer, PARP inhibitors, bevacizumab

## Abstract

**Objective:**

As one of the cancers that seriously threatens women’s health, ovarian cancer has a high morbidity and mortality rate. Surgery and chemotherapy are the basic treatment strategies for ovarian cancer, and chemotherapy resistance is a significant factor in affecting the prognosis, survival cycle, and recurrence of ovarian cancer. This article aims to analyze articles about ovarian cancer and drug resistance via bibliometric software, offering new ideas and directions for researchers in this field.

**Methods:**

Both Citespace and Vosviewer are bibliometric software on the Java platform. Articles were collected on ovarian cancer and drug resistance in the Web of Science Core Collection database from 2013 to 2022. The countries, institutions, journals, authors, keywords, and references were analyzed, and the development status of this field was indicated from multiple perspectives.

**Results:**

Studies on ovarian cancer and drug resistance generally showed an increasing trend from 2013 to 2022. The People’s Republic of China and Chinese institutions contributed more to this field. *Gynecologic Oncology* published the most articles, and the journal with the most citations was *Cancer Research*. Li Li was the author with the most publications, and Siegel RL was the author with the most citations. Through burst detection, it can be found that the research hotspots in this field mainly focused on the in-depth exploration of the drug resistance mechanism of ovarian cancer and the progress of PARP inhibitors and bevacizumab in the treatment of ovarian cancer.

**Conclusions:**

Many studies on the mechanism of drug resistance in ovarian cancer have been discovered; however, the deeper mechanism remains to be explored. Compared with traditional chemotherapy drugs, PARP inhibitors and bevacizumab have shown better efficacy, but PARP inhibitors have initially shown drug resistance. The future direction of this field should be to overcome the resistance of existing drugs and actively develop new ones.

## 1 Introduction

According to relevant statistics, ovarian cancer ranks eighth among female tumors in terms of morbidity and mortality (1). In 2020, compared with 2018, the mortality of ovarian cancer increased by 0.3 percentage points (1, 2). In the estimated number of new cancer cases and deaths in the United States in 2022, ovarian cancer ranked fifth (3). All in all, ovarian cancer poses a serious threat to women’s health (4). As more attention is paid to ovarian cancer, various therapies have emerged, but drug resistance has also become increasingly common (5). Consequently, finding ways to address drug resistance is crucial for effective treatment.

Drug resistance involves many complex mechanisms. A large number of studies have shown that drug resistance in ovarian cancer may be mainly related to apoptosis inhibition pathways (6), the multidrug resistance gene (MDR) (7), non-coding RNA (8), the tumor microenvironment (9), and other factors related. Furthermore, different treatment methods can also generate corresponding drug resistance mechanisms. Khan et al. summarized the resistance mechanism of platinum drugs and came to the conclusion that most genes implicated in platinum resistance belong to DNA damage repair machinery (10). Gogola et al. found that loss of PAR glycohydrolase (PARG) is the major resistance mechanism of inhibitors of poly(ADP-ribose)(PAR) polymerase (PARPi) in the treatment of ovarian cancer (11). With more in-depth research on the mechanism of drug resistance in ovarian cancer, additional mechanisms are being gradually uncovered. Understanding these mechanisms is of great significance for clinical treatment and improving the prognosis of ovarian cancer.

The drug resistance mechanism of ovarian cancer is not yet fully disclosed, which can cause difficulties for researchers studying in this field. Fortunately, bibliometrics, as a recently emerging discipline, can analyze the contributions of countries/regions, institutions, authors, and journals in a certain field, as well as understand research hotspots and emerging keywords in this field. Furthermore, bibliometrics can predict future development prospects (12). Citespace and VOSviewer are Java-based bibliometric visualization analysis software that we used to analyze literature on ovarian cancer and drug resistance from 2013 to 2022 to identify the latest results and research hotspots in this field. Meanwhile, we provided reference directions for relevant researchers to avoid polyisomerism and improve scientific research efficiency (13, 14).

## 2 Materials and methods

### 2.1 Data collection

All documents in this bibliometric analysis were downloaded from the WOSCC (Web of Science Core Collection). Compared with other related databases, the data in WOS are more representative and influential. Moreover, studies have shown that the results of bibliometric analysis using the data in WOS are better visualized (15, 16).

The search formula used in this study was TI = ((resist*) OR (drug resist*) OR (medicine resist*) OR (agent resist*) OR (Antineoplastic Agent resist*) OR (Antineoplastic Drug resist*) OR (Antineoplastic resist*)) AND TI = ((Ovarian Cancer*) OR (Ovarian Carcinoma) OR (Ovarian Neoplasm*) OR (Cancer of Ovary) OR (Cancer of the Ovary)). To improve the data time sensitivity, the post date was limited to 2013–2022. Article and review were set as the document types and English as the language type. Data collection was completed on 8 November 2022 and saved as a txt file in the Full Record and Cited References format. A total of 1,511 documents were obtained for the next visual analysis.

### 2.2 Data analysis

Microsoft Excel (version 2019), Citespace (6.1.R3), and VOSviewer (1.6.18) were used for data analysis. Microsoft Excel 2019 was used to analyze the annual trend in the number of articles published. The content of the analysis included the contributions of countries/regions, institutions, authors, and journals in this field, as well as the clustering and burst of keywords and co-cited references. The node size indicated the frequency of occurrence or reference, while the connection between the nodes indicated the strength of the connection. In addition, we also calculated the betweenness centrality of the node to determine its significance and whether it served as a bridge role (17). In keyword clustering, the modularity Q and the mean silhouette scores reflect the clustering situation. Generally speaking, when Q >0.3, the clustering structure is significant, while when S >0.5, the clustering is reasonably significant (18). VOSviewer was used to analyze the related situation in journals. Compared with Citespace, its density map showed the distribution of journals better (14).

## 3 Result

### 3.1 Annual growth trends of publication

**Figure 1:**
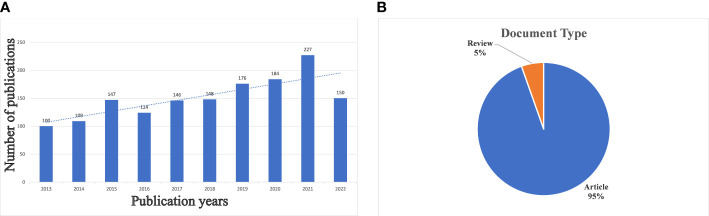
**(A)** Annual growth trends of publication. The number of publications in 2013–2022 was an increasing trend. **(B)** The distribution of document type. Article accounts for 95% and review accounts for 5%.

To investigate the research status in ovarian cancer and drug resistance, we utilized Microsoft Office 2019 software to count the number of papers published in each year from 2013 to 2022 and plotted the results on a trend line. As shown in **Figure 1A**, generally, there has been an upward trend in the number of publications in the past decade, which indicates drug resistance issues in ovarian cancer have become increasingly important topics. In 2021, the number of publications increased by 43, which was the largest expansion compared with 2020. Moreover, the growth of the trend line suggests that this field of study still presents promising research opportunities. In terms of article types (**Figure 1B**), articles accounted for a large part (95%), while reviews made up a smaller portion (5%).

### 3.2 Collaborative networks of countries/regions and institutions co-occurrence

The 1,511 articles encompassed a total of 58 countries/regions and 363 research institutions. The number of co-occurrences between countries was 2,064, while the number of co-occurrences between institutions was 1,676. As shown in **Table 1**, the People’s Republic of China ranked first with 728 contributions. The top ten institutions, due to the relatively high number of contributions, were all from the People’s Republic of China.

**Table 1:** The top 15 co-occurrences numbers of countries/regions and institutions related to ovarian cancer drug resistance.

Rank	Country/Region	Frequency	Year	Centrality	Institution	Frequence	Year	Centrality
1	People’s Republic of China	728	2013	0.03	China Med Univ	53	2013	0.06
2	USA	401	2013	0.23	Fudan Univ	45	2013	0.08
3	Italy	82	2013	0.08	Zhejiang Univ	42	2013	0.05
4	England	77	2013	0.25	Huazhong Univ Sci & Technol	34	2013	0.09
5	Japan	71	2013	0.02	Zhengzhou Univ	29	2013	0.07
6	Canada	62	2013	0.14	Harbin Med Univ	28	2014	0.02
7	South Korea	62	2013	0.01	Shandong Univ	28	2015	0.04
8	Germany	60	2013	0.17	Guangxi Med Univ	27	2013	0
9	Australia	57	2013	0.14	Shanghai Jiao Tong Univ	26	2013	0.02
10	France	41	2013	0.09	Jilin Univ	26	2013	0.02
11	Poland	41	2013	0	Poznan Univ Med Sci	25	2013	0.03
12	Spain	37	2014	0.07	Univ Texas MD Anderson Canc Ctr	23	2013	0.13
13	Belgium	27	2013	0.06	Nanjing Med Univ	23	2013	0.01
14	Iran	24	2015	0	Capital Med Univ	20	2014	0.01
15	Denmark	22	2013	0.03	Sun Yat Sen Univ	20	2017	0.07

**Figure 2:**
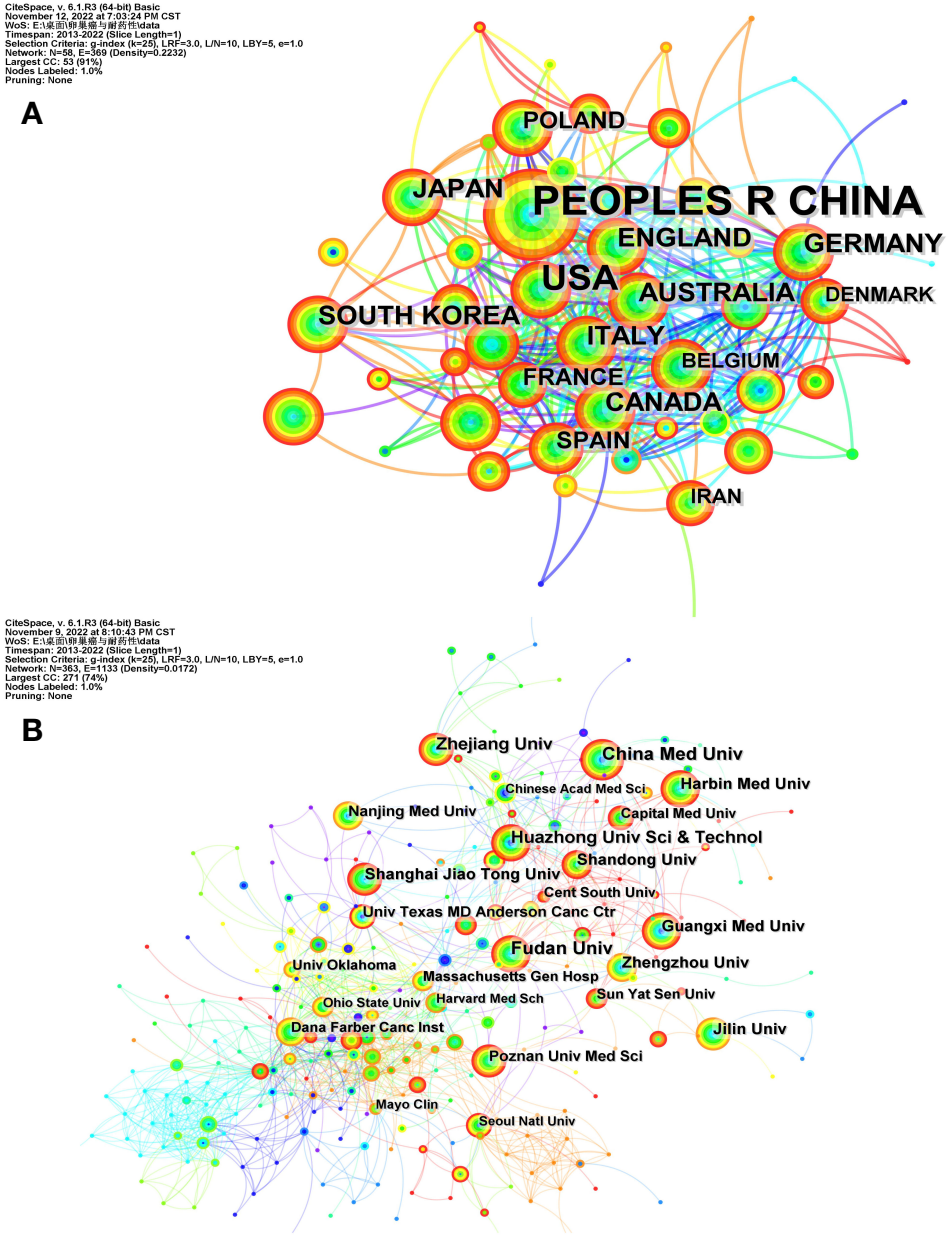
The co-occurrence of cooperation networks of countries/regions **(A)** and institutions **(B)** related to ovarian cancer drug resistance. The nodes represent countries/regions or institutions. The size of nodes represents the number of published documents, the more published documents, the larger the nodes. The color of each layer of nodes represents the year of publication. The connection between nodes represents the cooperation between countries/regions or institutions, the density of the lines represents the level of the combinations. The color of the connecting line represents the cooperation time.

The betweenness centrality of England and the USA was 0.25 and 0.23, respectively, which demonstrated that these two countries played a crucial role as bridges in the studies conducted in this field. Among institutions, the contribution of the University of Texas MD Anderson Cancer Center was not much, but its betweenness centrality was 0.13, significantly higher than other institutions, which indicated it played a vital role in the cooperation of institutions. **Figure 2** illustrates the co-occurrence of cooperation networks among countries/regions and institutions.

### 3.3 Analysis of journals and co-cited journals

**Figure 3:**
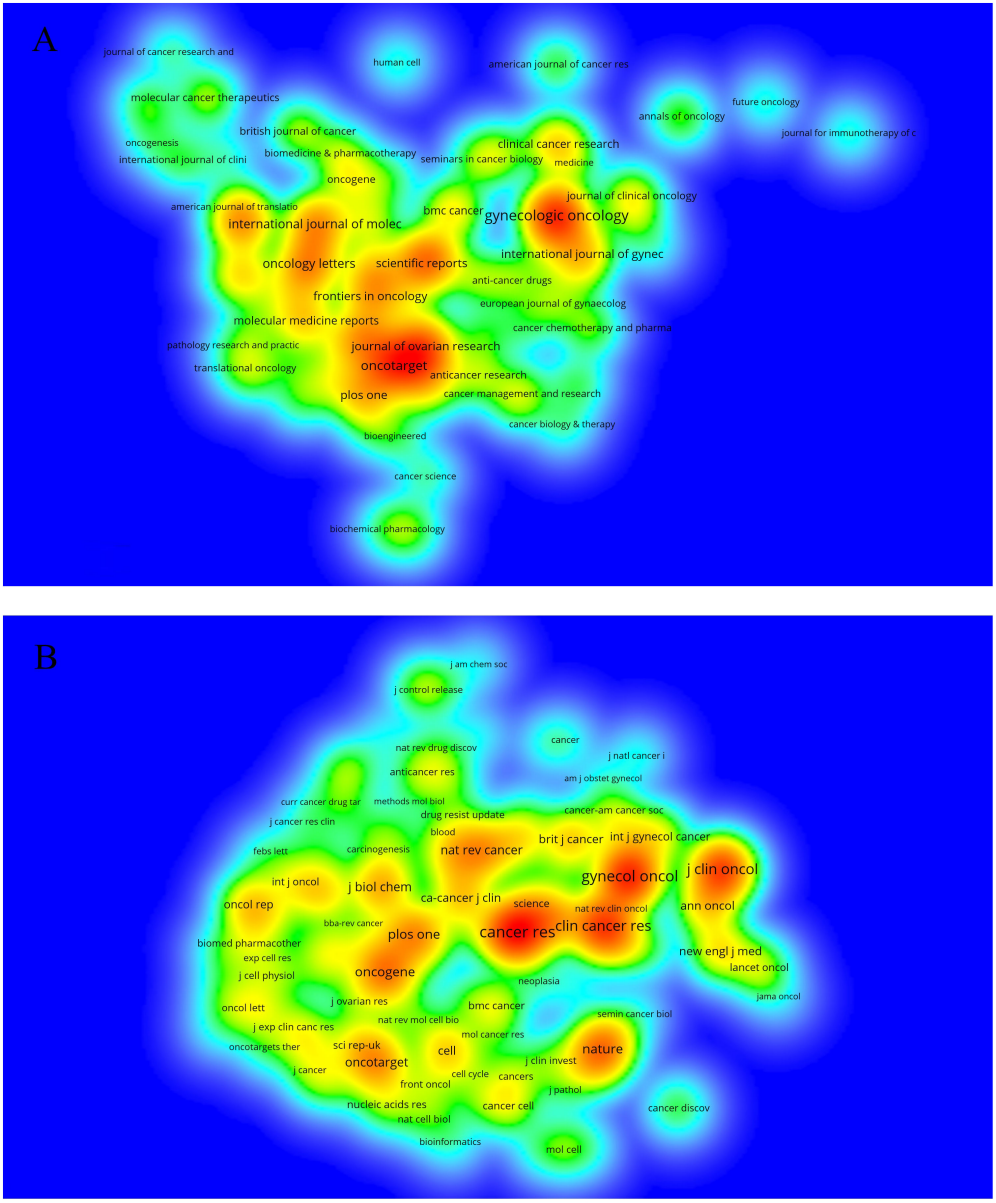
The density map of journals **(A)** and co-cited journals **(B)** related to ovarian cancer drug resistance. The title of the journal is marked on the figure. The more publications or citations, the closer to red. **(A)** the number of publications ≥5; **(B)** the number of journals citations ≥100.

These 1,511 articles were published in 404 journals from 2013 to 2022. The top ten journals with the most publications are listed in **Table 2**. The density map in **Figure 3A** shows the publication volume of each journal. “*Gynecologic Oncology*,” the top journal, had published 68 articles, and the impact factor of this journal had increased to 5.304. Other high-volume journals were all related to tumors, including “*Oncotarget*,” “*International Journal of Molecular Sciences*,” “*Oncology Letters*,” and “*Oncology R*.” The impact factors were between 3 and 7, so, these journals had a very important position.

**Table 2:** The top 10 journals and co-cited journals related to ovarian cancer drug resistance.

Rank	Journal	Count	If (2021)	Co-cited Journal	Citation	If (2021)
1	Gynecologic Oncology	68	5.304	Cancer Research	2,585	13.312
2	Oncotarget	43	5.168(2016)	Gynecologic Oncology	2,339	5.304
3	International Journal of Molecular Sciences	37	6.208	Journal of Clinical Oncology	2,018	50.739
4	Oncology Letters	36	3.111	Clinical Cancer Research	1,816	13.801
5	Oncology Reports	34	4.136	Oncogene	1,442	8.756
6	Journal of Ovarian Research	29	5.506	PLOS One	1,220	3.752
7	Scientific Reports	29	4.997	Nature	1,216	69.504
8	International Journal of Oncology	26	5.884	Oncotarget	1,157	5.168 (2016)
9	International Journal of Gynecological Cancer	26	4.661	Nature Reviews Cancer	1,078	69.800
10	PLOS One	25	3.752	Proceedings of the National Academy of Sciences of the United States of America	1,008	12.779

To investigate the journal articles that researchers preferred to cite and reflect the source of ovarian cancer and drug resistance knowledge, we analyzed the journal co-citations. The top ten journals with the most citations are listed in **Table 2**. Most of these journals were related to clinical tumor research. **Figure 3B** provides a density plot indicating that the most cited journal was “*Cancer Research*” with 2,585 citations and a high impact factor of 13.512. Among other journals, “*Nature Reviews Cancer*” had 1,078 citations, which played an essential role in the source of knowledge with an impact factor of 69.800 in this field.

### 3.4 Analysis of authors and co-cited authors

**Figure 4:**
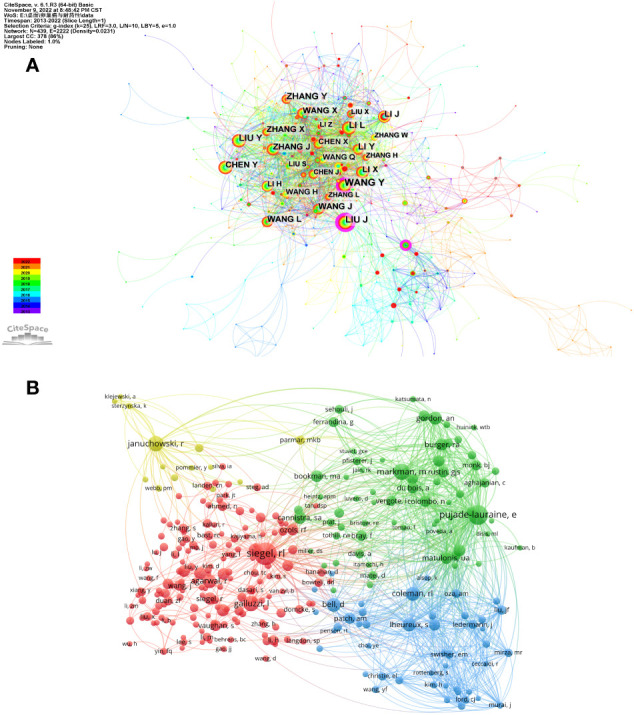
The visualization of author’s cooperation network **(A)** and co-cited author network **(B)** related to ovarian cancer drug resistance. **(A)** Each node represents an author. The size of node indicates how much the author has published. The lines between authors reflect their collaborative relationships. Authors with purple circles indicate that their betweenness centrality is high (≥0.2), and they have a certain bridge effect. **(B)** Each node represents an author. The size of a node represents the number of references. Author descriptions of the same node color are a small group. Authors with citations ≥20 are shown in the figure.

A total of 8,938 authors participated in the writing of these 1,511 articles, and relevant information is shown in **Table 3**. It can be observed that Li Li published the highest number of articles, with a total of 30 publications. Januchowski Radoslaw, Yin Fuqiang, Nowicki Michal, Sterzynska Karolina, and Zhang Wei also published more than 15 articles. Nevertheless, the betweenness centrality of the top ten authors was relatively low. As shown in **Figure 4A**, the cooperation network among authors was very intensive. Interestingly, the top ten authors by number of publications were disadvantaged in the cooperation network. Wang Yu and Liu Juanjuan, who had published 7 and 5 papers, respectively, had betweenness centralities of 0.2 and 0.27, becoming the “bridge” in the authors’ cooperation network.

**Table 3:** The top 10 authors and co-cited authors related to ovarian cancer drug resistance.

Rank	Author	Count	Centrality	Co-cited Author	Citation	Centrality
1	Li Li	30	0.03	Siegel RL	346	0
2	Januchowski Radoslaw	23	0	Pujade-Lauraine E	257	0.04
3	Yin Fuqiang	19	0	Markman M	175	0.02
4	Nowicki Michal	18	0	Januchowski R	161	0.03
5	Sterzynska Karolina	16	0	Galluzzi L	158	0.03
6	Zhang Wei	15	0.03	Bell D	131	0.05
7	Zabel Maciej	13	0	Coleman RL	125	0.05
8	Liu Xia	12	0.01	Agarwal R	123	0.03
9	Moore Kathleen N.	12	0.04	Lheureux S	121	0
10	Swierczewska Monika	12	0	Gordon AN	112	0.02

Furthermore, an analysis of co-cited authors was carried out, with the results shown in **Figure 4B**. Siegel RL, became the first co-cited author with 346 citations. Among the top five, Pujade-Lauraine E, Markman M, Januchowski R, and Galluzzi L all had more than 150 citations. But their betweenness centrality was not high enough.

### 3.5 Keyword co-occurrence, cluster, and burst detection

**Figure 5:**
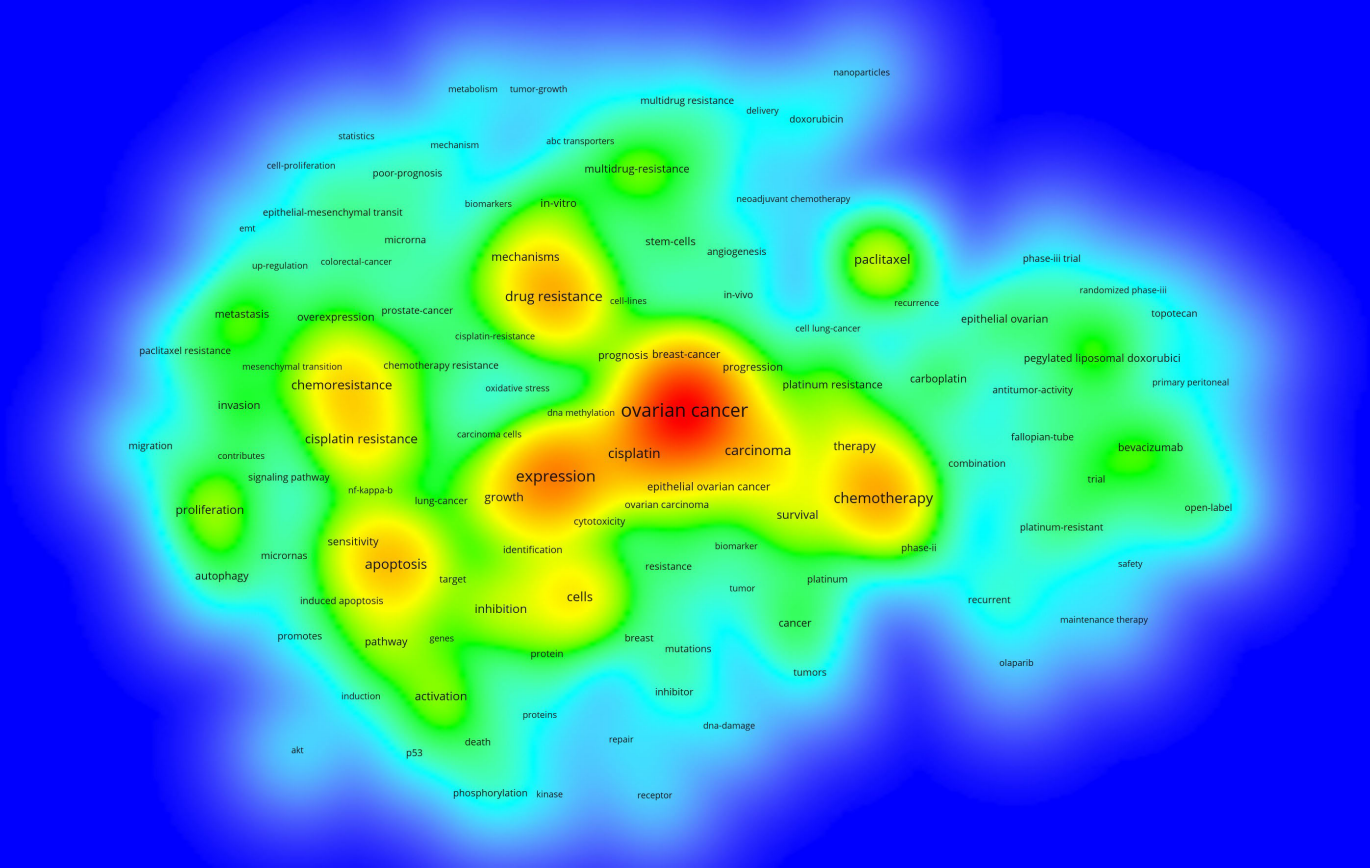
The density map of keyword co-occurrence related to ovarian cancer drug resistance. The higher the keyword frequency, the closer to red. Minimum number of occurrences of keywords ≥20.

Keywords can effectively reflect the research hotspots and directions in a specific field. First, we used VOSviewer to analyze the co-occurrence of keywords in this domain. The top 20 keywords with the highest frequency of co-occurrence are listed in **Table 4** after merging duplicate-meaning keywords such as drug-resistance and drug resistance. The analysis revealed that ovarian cancer (n = 813) had the highest frequency, followed by expression (n = 406), chemotherapy (n = 310), drug resistance (n = 281), and cisplatin (n = 259). Additionally, the distribution of each keyword is visualized in the density map in **Figure 5**.

**Table 4:** The top 20 keywords related to ovarian cancer drug resistance.

Rank	Keyword	Count	Rank	Keyword	Count
1	ovarian cancer	813	11	paclitaxel	165
2	expression	406	12	growth	134
3	chemotherapy	310	13	proliferation	123
4	drug resistance	281	14	survival	121
5	cisplatin	259	15	therapy	120
6	apoptosis	244	16	inhibition	116
7	carcinoma	220	17	activation	101
8	cells	195	18	mechanisms	101
9	chemoresistance	182	19	breast-cancer	100
10	cisplatin resistance	173	20	multidrug-resistance	100

**Figure 6:**
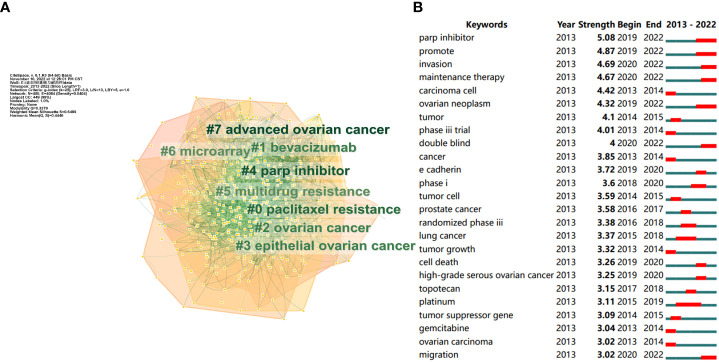
**(A)** The clusters of keywords related to ovarian cancer drug resistance. Through keyword clustering, keywords in the same field can be analyzed together. **(B)** The top 25 keywords with burst strength related to ovarian cancer drug resistance. The order of keywords is arranged according to burst strength. The larger the burst strength, the higher the popularity of the keyword.

Then, Citespace was used to cluster the keywords, and the outcomes were displayed in **Figure 6A**. All keywords were clustered into eight categories, including paclitaxel resistance (Cluster 0), multidrug resistance (Cluster 5), and parp inhibitor (Cluster 4) related to drug resistance. Furthermore, ovarian cancer (Cluster 2), epithelial ovarian cancer (Cluster 3), and advanced ovarian cancer (Cluster 7) were related to ovarian cancer classification, microarray (Cluster 6) was related to ovarian cancer research technology, and bevacizumab (Cluster 1) was associated with the treatment of ovarian cancer. Keyword clustering can well represent research branches and hotspots in this field. Meanwhile, we performed keyword burst detection. **Figure 6B** depicts the top 25 keywords with burst strength, in which “parp inhibitor” (5.08) had the highest burst strength. “Promote” (4.87), “invasion” (4.69), and “maintenance therapy” (4.67), all exceeding 4.5, reflect the research frontier to some extent.

**Figure 7:**
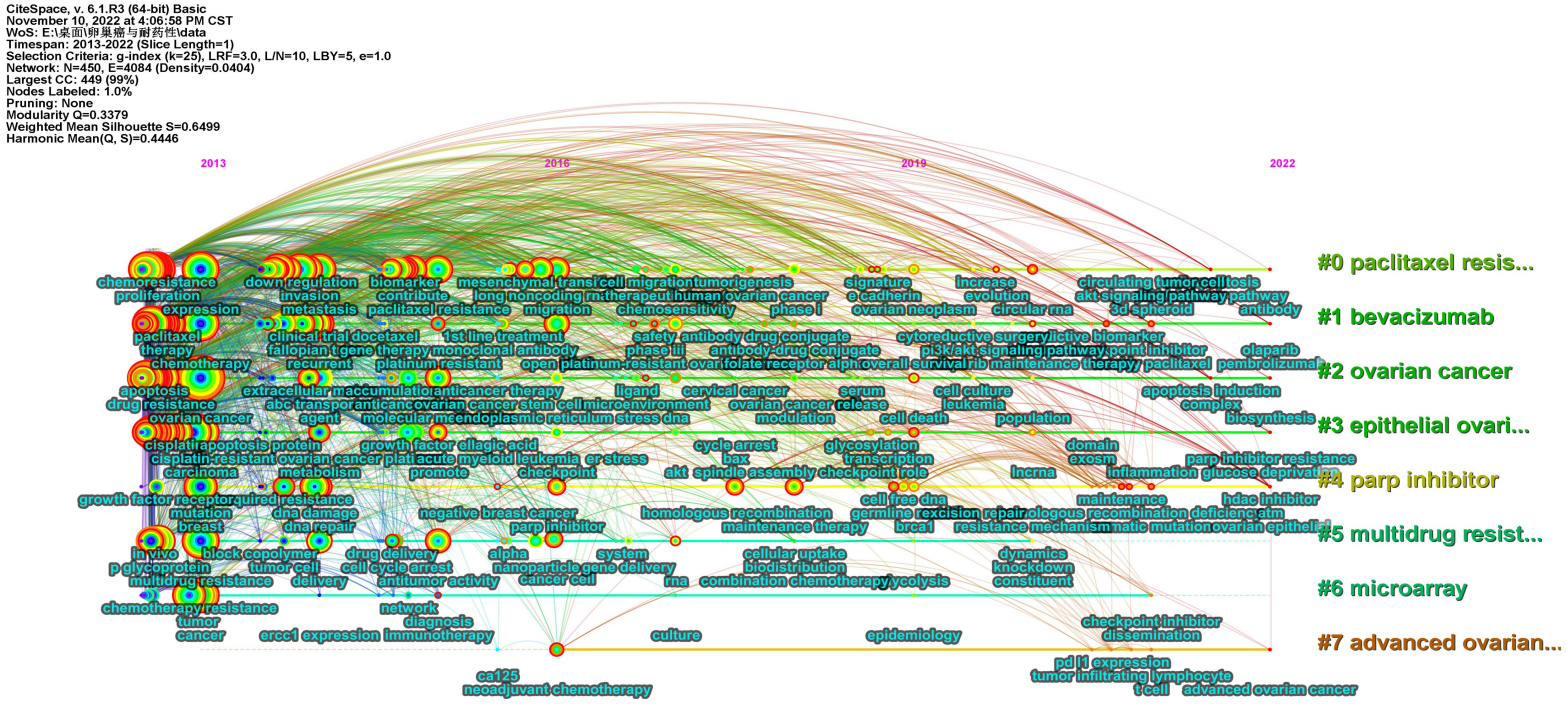
Timeline analysis of the keywords related to ovarian cancer drug resistance. Each keyword cluster has its own timeline. Each node represents a keyword, and the keywords are marked under the node. The order of appearance of the nodes indicates the development and evolution process of the keywords under the cluster.

To investigate the development and evolution processes and the research trend, we conducted a timeline analysis of keywords. As shown in **Figure 7**, paclitaxel resistance (Cluster 0), bevacizumab (Cluster 1), ovarian cancer (Cluster 2), epithelial ovarian cancer (Cluster 3), and multidrug resistance (Cluster 5) started earlier. Despite the fact that advanced ovarian cancer (Cluster 7) started late, it had a tendency to catch up later. Bevacizumab (Cluster 1) and parp inhibitors (Cluster 4) have maintained high popularity in recent years.

### 3.6 Analysis of references and co-cited references

Co-cited references refer to documents commonly cited in papers in this field and can also reflect the source of knowledge in this field. VOSviewer was used to count the top 10 articles with the most citations. As shown in **Table 5**, the article with the most citations was “Bevacizumab combined with chemotherapy for platinum-resistant recurrent ovarian cancer: the AURELIA open-label randomized phase III trial” (19), which was published by Pujade-Lauraine E in the *Journal of Clinical Oncology* in 2014 and had 148 citations. It is observed that the journal sources of the top 10 most cited articles were from the *Journal of Clinical Oncology*, *Nature*, *Nature Reviews Cancer*, *Oncogene*, *Lancet*, and *CA-A Cancer Journal for Clinicians*, with two articles each.

**Table 5:** The top 10 articles with the most citations related to ovarian cancer drug resistance.

Rank	Title	Author	Year	Journal	Citation
1	Bevacizumab combined with chemotherapy for platinum-resistant recurrent ovarian cancer: the AURELIA open-label randomized phase III trial	Pujade-lauraine E	2014	Journal of Clinical Oncology	148
2	Integrated genomic analyses of ovarian carcinoma	Bell D	2011	Nature	130
3	Ovarian cancer: strategies for overcoming resistance to chemotherapy	Agarwal R	2003	Nature Reviews Cancer	120
4	Molecular mechanisms of cisplatin resistance	Galluzzi L	2012	Oncogene	112
5	Ovarian cancer	Jayson GC	2014	Lancet	98
6	Cancer statistics, 2014	Siegel RL	2015	CA-A Cancer Journal for Clinicians	93
7	Whole–genome characterization of chemoresistant ovarian cancer	Patch AM	2015	Nature	87
8	Rethinking ovarian cancer: recommendations for improving outcomes	Vaughan S	2011	Nature Reviews Cancer	79
9	Randomized Phase III Trial of Gemcitabine Compared with Pegylated Liposomal Doxorubicin in Patients with Platinum-Resistant Ovarian Cancer	Mutch DG	2007	Journal of Clinical Oncology	75
10	Global cancer statistics 2018: GLOBOCAN estimates of incidence and mortality worldwide for 36 cancers in 185 countries	Bray F	2018	CA-A Cancer Journal for Clinicians	69

**Figure 8:**
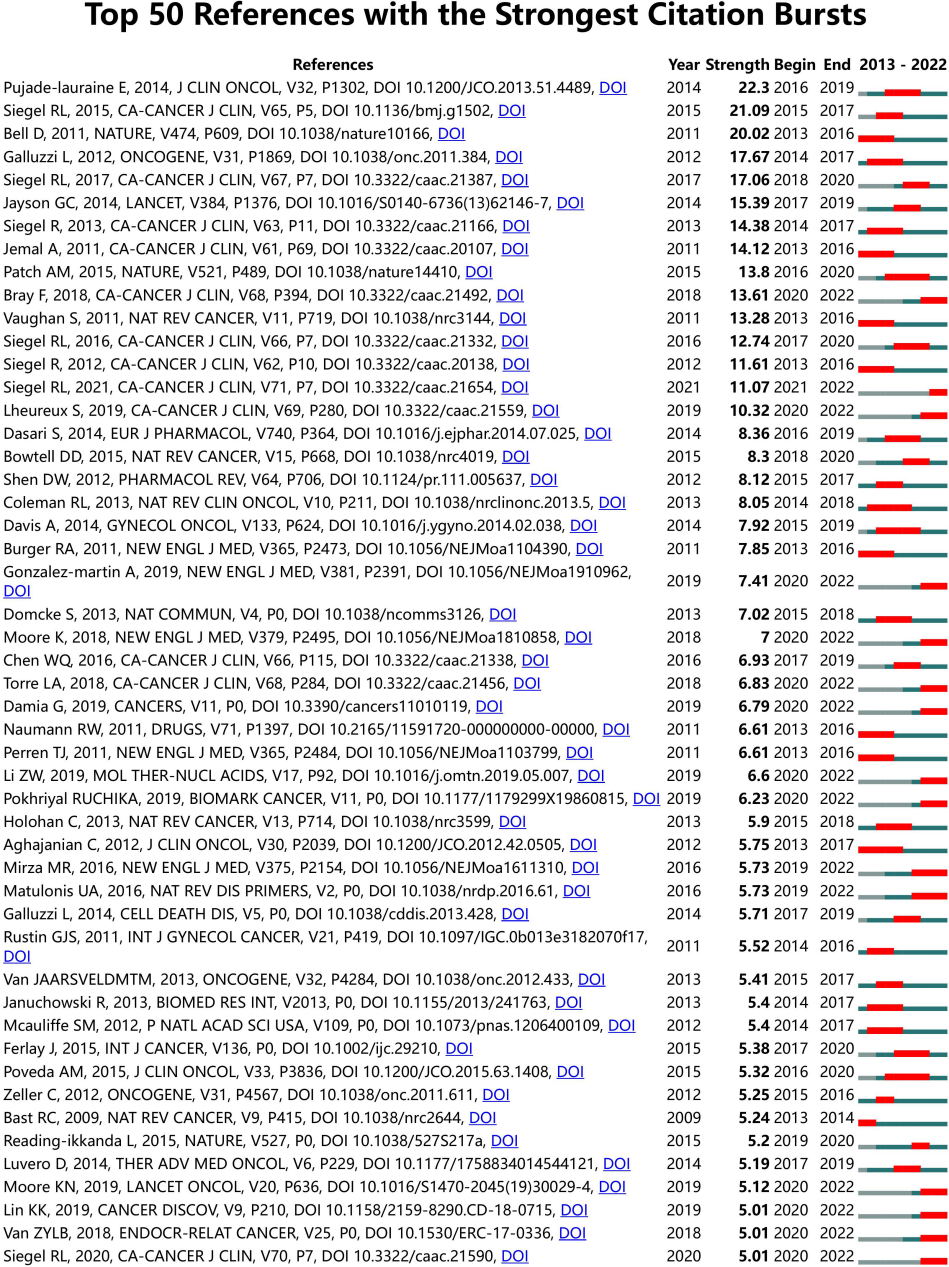
The top 50 references with burst strength related to ovarian cancer drug resistance. The higher the burst strength, the higher the heat.

Furthermore, Citespace was employed to conduct burst analysis on the references, and the top 50 references with burst strengths are displayed in **Figure 8**. It is noteworthy that the author, Siegel R (L), contributed seven articles that mainly contained statistical cancer data for a particular year, laying the groundwork for the study of ovarian cancer and drug resistance.

**Figure 9:**
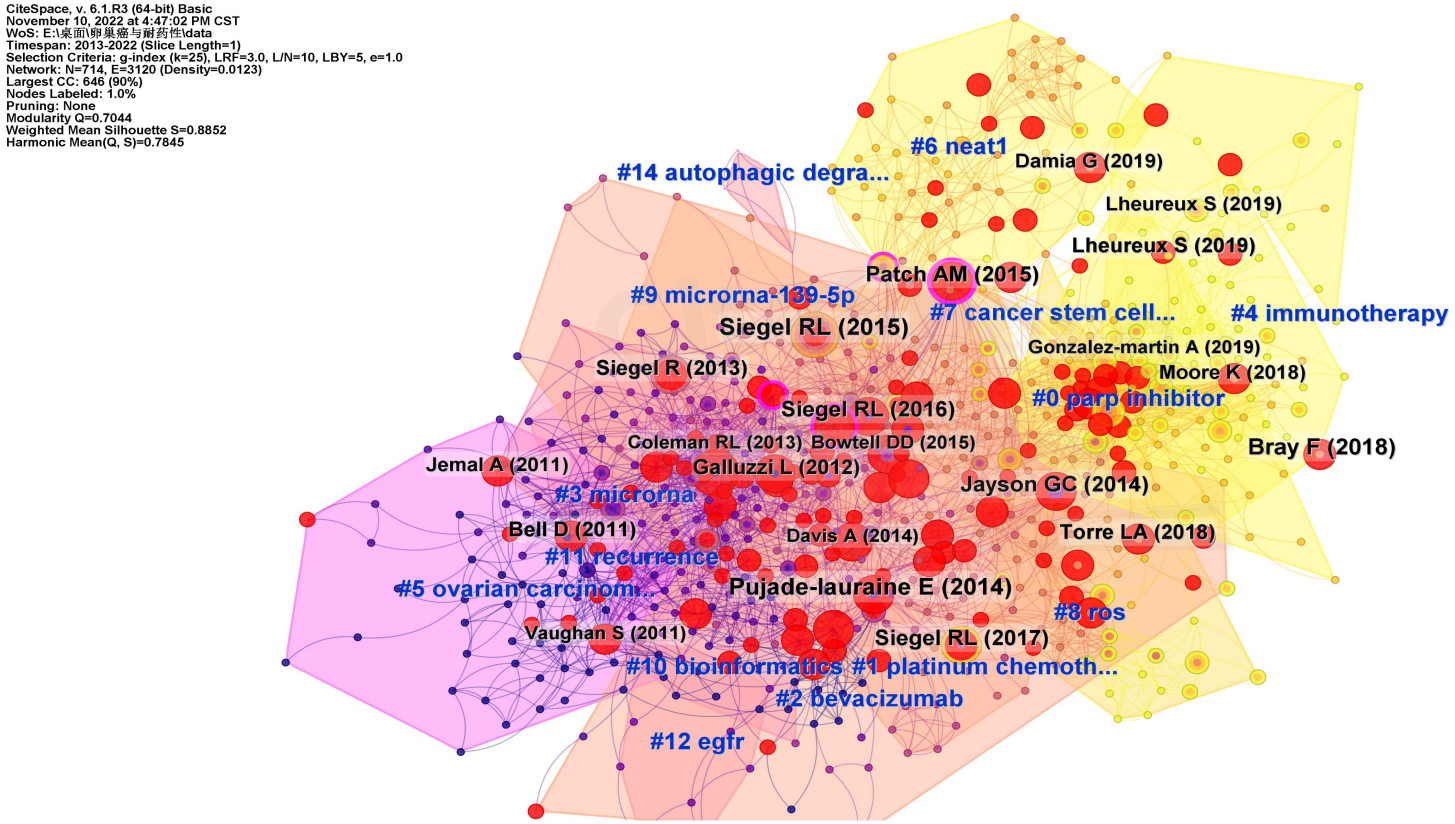
The clusters of references related to ovarian cancer drug resistance. Documents of the same field or type are clustered together. The color of the clusters ranged from purple to orange to yellow, indicating changes in references over time.

In addition, we also used Citespace to cluster the references, as shown in **Figure 9**. All references were clustered into 15 categories. This approach enabled better classification of the references and made it easier to understand the fields and categories of the references.

## 4 Discussion

### 4.1 General information

A total of 1,511 documents were collected for this study. The analysis of the annual publication volume reveals that research on ovarian cancer and drug resistance has been continuously increasing from 2013 to 2021, and the publication volume is expected to continue to grow in 2022. This trend may be related to the emergence of ovarian cancer drug resistance and the increasing harm of ovarian cancer to women.

### 4.2 Contributions by countries, institutions, journals, and authors

In all countries involved, the People’s Republic of China ranked first, with all the top ten institutions from China. This demonstrates the important position of China in the field of drug resistance in ovarian cancer.

Relevant epidemiological surveys and studies have shown that in the past decade, the incidence of ovarian cancer in China has shown an upward trend. Although the annual age-standardized incidence rate of ovarian cancer in China was lower than that of the world, South Korea, Japan, and Singapore, it has shown a continuous growth trend over the past 30 years, and the increase rate exceeded that of the world, Japan, and Singapore (20). These could be one of the reasons why China has done extensive research in this field. China recently did a lot of work in the discovery of multiple drug resistance mechanisms, including overexpression of the USP11-BIP axis leading to drug resistance (21), significantly upregulated TTK expression in high-grade serous ovarian carcinoma (HGSOC), and cisplatin-resistant ovarian cancer cells (22). These have enabled China to become a powerful nation in this field.

However, the betweenness centrality of the People’s Republic of China and Chinese institutions was slightly lower than others. While the betweenness centrality of England and the USA both exceeded 0.1, which played an important role as a bridge in this field. In the ranking of institutional contributions, China Medical University ranked first.

Among the top ten journals with article sources, four were from the USA, three were from England, and two were from Greece. These countries have played a certain role in promoting this field. Further observation of the number of published articles and the cooperation network of the authors showed that the cooperation between the authors was mainly concentrated among the Chinese, but the cooperation among other authors with many published articles was not very intensive.

It is worth mentioning that Siegel RL is a cancer epidemiologist and Senior Scientific Director of Surveillance Research at the American Cancer Society and updates cancer statistics every year. Although she topped the list with 346 citations, it may not have much to do with ovarian cancer and drug resistance since her research covers all types of cancer, not just ovarian cancer.

Continuing to observe the top ten authors in terms of the number of publications, we can find that they belong to two teams. The first group is from the Tumor Hospital of Guangxi Medical University, including Li Li, Yin Fuqiang, Zhang Wei, and Liu Xia. Their collaboration started in 2013 and continued into 2021. Their articles primarily investigated the impact of genes on ovarian cancer development and drug resistance by employing bioinformatics methods and utilizing relevant databases (23, 24). Their work is significant in guiding the direction of experimental research in ovarian cancer (25). The second group includes Januchowski Radoslaw, Nowicki Michal, Sterzynska Karolina, Swierczewska Monika, and Zabel Macie. Their articles are primarily focused on cellular experiments to explore the role of genes or proteins in the mechanism of drug resistance in ovarian cancer (26, 27). In addition, they also found that piperine may treat PAC- and TOP-resistant ovarian cancer (28). Generally speaking, both teams have contributed to investigating the relationship between genes and drug resistance in ovarian cancer.

### 4.3 Correlation analysis of keyword

Through keyword co-occurrence, clustering, and burst detection, we can find that the current research hotspots, and frontiers of ovarian cancer and drug resistance mainly focus on the following aspects.

#### 4.3.1 The impact of some specific genes or substances on the drug resistance of ovarian cancer

With the widespread use of chemotherapy drugs, drug resistance in ovarian cancer is becoming more common. Over 50% of patients with serous ovarian cancer who receive effective anticancer drug treatments relapse within five years and develop drug resistance (29). Many genes may improve the development of ovarian cancer, and their expression disorder will cause drug resistance in ovarian cancer cells (30). For instance, the p-glycoprotein (abc transporter ABCB1) encoded by the mdr1 gene in cells will lead to the formation of paclitaxel resistance (7). Multidrug resistance develops when ovarian cancer cells actively pump therapeutic drugs out of the cell through transporters (31).

#### 4.3.2 PARP inhibitors

PARP inhibitors are used to treat homologous recombination (HR) DNA repair-deficient tumors (e.g., BRCA1/2-mutated) (32), which was once used to treat breast cancer. In high-grade serous ovarian carcinoma (HGSOC), PARP inhibitors are particularly sensitive to HGSOC with mutations in BRCA1 or BRCA2 (BRCA1/2) (33). Therefore, it has also been used in the treatment of HGSOC in recent years. Studies also indicated that the use of PARP inhibitors (Olaparib) as first-line drugs can lower the risk of disease progression or death by up to 70% compared to their administration as third- or fourth-line treatments (34). Finally, the US Food and Drug Administration as well as the European Medicines Agency approved the use of niraparib (PARP inhibitors) for epithelial ovarian cancer, regardless of whether the BRCA gene was mutated, which greatly relaxed the indications (35, 36).

However, with the application of PARP inhibitors, secondary mutations in BRCA1/2 lead to the restoration of HR function (33). HR can repair double-strand DNA breaks (DSBs) and reduce cell death, and the problem of newly acquired drug resistance appears (37). The issue significantly impacts HGSOC treatment. A recent study has shown that combining PARP inhibitors with other drugs can improve the therapeutic effect of PARP inhibitors, and especially the combination with anti-angiogenic therapy may be evaluated most (38), which helped to address the problem of resistance to PARP inhibitors, but more study on other treatment methods is still necessary.

In the burst detection of keywords in our study, PARP inhibitors ranked first with a burst strength of 5.08. It appeared from 2019 to 2022, and it is likely to continue in the future. In general, PARP inhibitors are the current research hotspots for ovarian cancer and drug resistance.

#### 4.3.3 Bevacizumab

Bevacizumab is a fully humanized monoclonal antibody that can inhibit the activity of vascular endothelial growth factor and tumor angiogenesis to treat tumors (39, 40). Bevacizumab also has significant advantages in treating elderly patients with platinum-resistant recurrent ovarian cancer (41). A randomized phase III AURELIA trial was performed, which showed that adding bevacizumab to chemotherapy significantly improved progression-free survival and the overall response rate. Therefore, European and US regulatory authorities have approved bevacizumab for platinum-resistant OC (19), and the combination of bevacizumab and chemotherapy has solved the issue of drug resistance in ovarian cancer to a certain extent.

### 4.4 Correlation analysis of co-cited references

By analyzing the co-cited references with high citations, we can roughly identify the main knowledge sources in this field. In this study, we focused on the top four cited references to gain a general understanding of the topic. These four documents are considered highly representative, with more than 100 times cited.

The first title is “Bevacizumab combined with chemotherapy for platinum-resistant recurrent ovarian cancer: The AURELIA open-label randomized phase III trial” (19). This study introduces a novel method for treating platinum-resistant ovarian cancer by combining single-agent chemotherapy with biologic therapies, specifically the monoclonal antibody bevacizumab. It was finally concluded that this combination could significantly improve progression-free survival (PFS) through a rigorous randomized phase III trial.

The second title is “Integrated genomic analyses of ovarian carcinoma” (42). After analyzing the messenger RNA expression, microRNA expression, promoter methylation, and DNA copy number in the genome of 489 cases of high-grade serous ovarian cancer, the article obtained the characteristics of related genes in ovarian cancer and provided a direction for the selection of therapeutic targets for ovarian cancer.

The third title is “Ovarian cancer: strategies for overcoming resistance to chemotherapy” (43), which was published in 2003. This article mainly reviewed the treatment methods for ovarian cancer, in which paclitaxel and platinum chemotherapy are the basic treatments. The mechanisms of drug resistance and possible treatment methods were also proposed.

The last title is “Molecular mechanisms of cisplatin resistance” (44). This article systematically expounded the resistance mechanism of cisplatin in the treatment of ovarian cancer and divided it into four types, including the steps preceding the binding of cisplatin to DNA (pre-target resistance), directly related to DNA-cisplatin adducts (on-target resistance), the lethal signaling pathways elicited by cisplatin-mediated DNA damage (post-target resistance), and molecular circuits that do not present obvious links with cisplatin-elicited signals (off-target resistance). Finally, the article proposed combined strategies that can be used to reverse tumor cisplatin resistance. Although cisplatin has certain side effects, it’s still an important means of treating solid tumors. This article pointed the way to overcoming cisplatin resistance for many researchers.

## 5 Limitations

Despite our comprehensive analysis of articles on ovarian cancer and drug resistance over the decade from 2013 to 2022, there are still some limitations. Firstly, articles were collected on 8 November 2022, but since the year was not yet over, there is a possibility that articles were not included in the statistics after this time point. Secondly, we only collected articles from the Web of Science, which may cause the omission of articles from other repositories. Thirdly, we only screen articles and reviews in the English language, which may exclude articles with high academic impact.

## 6 Conclusion

In summary, the research on ovarian cancer and drug resistance has generally shown an increasing trend from 2013 to 2022. The People’s Republic of China and Chinese institutions have done more research in this field. In the future study, the relationship and cooperation between countries and authors should be continually strengthened. The research hotspots in this field mainly focus on the in-depth exploration of the drug resistance mechanism in ovarian cancer and the progress of PARP inhibitors and bevacizumab in ovarian cancer.

## Author contributions

LinZ was responsible for developing concepts and directing projects. JL, JZ, CL, and BY were responsible for literature search and data collection. HC, KD, and LiuZ were responsible for data analysis and collation. JL was responsible for data visualization analysis and article writing. JM was responsible for critical revision and proofreading of the article. All authors contributed to the article and approved the submitted version.

## References

[1] Sung H Ferlay J Siegel RL Laversanne M Soerjomataram I Jemal A. Global cancer statistics 2020: GLOBOCAN estimates of incidence and mortality worldwide for 36 cancers in 185 countries. CA Cancer J Clin (2021) 71:209–49. doi: 10.3322/caac.21660 33538338

[2] Bray F Ferlay J Soerjomataram I Siegel RL Torre LA Jemal A. Global cancer statistics 2018: GLOBOCAN estimates of incidence and mortality worldwide for 36 cancers in 185 countries. CA Cancer J Clin (2018) 68:394–424. doi: 10.3322/caac.21492 30207593

[3] Siegel RL Miller KD Fuchs HE Jemal A. Cancer statistics, 2022. CA Cancer J Clin (2022) 72:7–33. doi: 10.3322/caac.21708 35020204

[4] Chen J Wei Z Fu K Duan Y Zhang M Li K. Non-apoptotic cell death in ovarian cancer: treatment, resistance and prognosis. BioMed Pharmacother (2022) 150:112929. doi: 10.1016/j.biopha.2022.112929 35429741

[5] Norouzi-Barough L Sarookhani MR Sharifi M Moghbelinejad S Jangjoo S Salehi R. Molecular mechanisms of drug resistance in ovarian cancer. J Cell Physiol (2018) 233:4546–62. doi: 10.1002/jcp.26289 29152737

[6] Yuan J Lan H Jiang X Zeng D Xiao S. Bcl−2 family: novel insight into individualized therapy for ovarian cancer (Review). Int J Mol Med (2020) 46:1255–65. doi: 10.3892/ijmm.2020.4689 PMC7447322 32945348

[7] Tanaka K Kiguchi K Mikami M Aoki D Iwamori M. Involvement of the MDR1 gene and glycolipids in anticancer drug-resistance of human ovarian carcinoma-derived cells. Hum Cell (2019) 32:447–52. doi: 10.1007/s13577-019-00261-5 31350703

[8] Zhang C Wang M Shi C Shi F Pei C. Long non-coding RNA Linc00312 modulates the sensitivity of ovarian cancer to cisplatin via the bcl-2/Caspase-3 signaling pathway. Biosci Trends (2018) 12:309–16. doi: 10.5582/bst.2018.01052 29952351

[9] Yan H Guo B-Y Zhang S. Cancer-associated fibroblasts attenuate cisplatin-induced apoptosis in ovarian cancer cells by promoting STAT3 signaling. Biochem Biophys Res Commun (2016) 470:947–54. doi: 10.1016/j.bbrc.2016.01.131 26826383

[10] Khan MA Vikramdeo KS Sudan SK Singh S Wilhite A Dasgupta S. Platinum-resistant ovarian cancer: from drug resistance mechanisms to liquid biopsy-based biomarkers for disease management. Semin Cancer Biol (2021) 77:99–109. doi: 10.1016/j.semcancer.2021.08.005 34418576 PMC8665066

[11] Gogola E Duarte AA de Ruiter JR Wiegant WW Schmid JA de Bruijn R. Selective loss of PARG restores PARylation and counteracts PARP inhibitor-mediated synthetic lethality. Cancer Cell (2018) 33:1078–1093.e12. doi: 10.1016/j.ccell.2018.05.008 29894693

[12] Chen C. Searching for intellectual turning points: progressive knowledge domain visualization. Proc Natl Acad Sci U.S.A. (2004) 101 Suppl 1:5303–10. doi: 10.1073/pnas.0307513100 PMC387312 14724295

[13] Chen C Song M. Visualizing a field of research: a methodology of systematic scientometric reviews. PloS One (2019) 14:e0223994. doi: 10.1371/journal.pone.0223994 31671124 PMC6822756

[14] van Eck NJ Waltman L. Software survey: VOSviewer, a computer program for bibliometric mapping. Scientometrics (2010) 84:523–38. doi: 10.1007/s11192-009-0146-3 PMC2883932 20585380

[15] Me F Ei P Malietzis GA. Comparison of PubMed, scopus, web of science, and Google scholar: strengths and weaknesses. FASEB J (2008) 22:338–42. doi: 10.1096/fj.07-9492LSF 17884971

[16] Martín-Martín A Orduna-Malea E Thelwall M Delgado López-Cózar E. Google Scholar, web of science, and scopus: a systematic comparison of citations in 252 subject categories. J Informetrics (2018) 12:1160–77. doi: 10.1016/j.joi.2018.09.002

[17] Luo H Cai Z Huang Y Song J Ma Q Yang X. Study on pain catastrophizing from 2010 to 2020: a bibliometric analysis via CiteSpace. Front Psychol (2021) 12:759347. doi: 10.3389/fpsyg.2021.759347 34975649 PMC8718514

[18] Chen B Shin S. Bibliometric analysis on research trend of accidental falls in older adults by using citespace–focused on web of science core collection (2010–2020). Int J Environ Res Public Health (2021) 18:1663. doi: 10.3390/ijerph18041663 33572483 PMC7916410

[19] Pujade-Lauraine E Hilpert F Weber B Reuss A Poveda A Kristensen G. Bevacizumab combined with chemotherapy for platinum-resistant recurrent ovarian cancer: the AURELIA open-label randomized phase III trial. J Clin Oncol (2014) 32:1302–8. doi: 10.1200/JCO.2013.51.4489 24637997

[20] Yang X Chen D Wang H Fan W Pan S Hu M. Analysis of prevalence, trends and prediction of ovarian cancer among women in China. J Chongqing Med Univ (2022) 47:1030–5. doi: 10.13406/j.cnki.cyxb.003104

[21] Zhu X Zhang Y Luo Q Wu X Huang F Shu T. The deubiquitinase USP11 promotes ovarian cancer chemoresistance by stabilizing BIP. Signal Transduct Target Ther (2021) 6:264. doi: 10.1038/s41392-021-00580-w 34257276 PMC8277857

[22] Qi G Ma H Li Y Peng J Chen J Kong B. TTK inhibition increases cisplatin sensitivity in high-grade serous ovarian carcinoma through the mTOR/autophagy pathway. Cell Death Dis (2021) 12:1135. doi: 10.1038/s41419-021-04429-6 34876569 PMC8651821

[23] Yin F Liu X Li D Wang Q Zhang W Li L. Bioinformatic analysis of chemokine (C-c motif) ligand 21 and SPARC-like protein 1 revealing their associations with drug resistance in ovarian cancer. Int J Oncol (2013) 42:1305–16. doi: 10.3892/ijo.2013.1819 23404140

[24] Yin F Liu X Li D Wang Q Zhang W Li L. Tumor suppressor genes associated with drug resistance in ovarian cancer (review). Oncol Rep (2013) 30:3–10. doi: 10.3892/or.2013.2446 23660957

[25] Yin F Liu L Liu X Li G Zheng L Li D. Downregulation of tumor suppressor gene ribonuclease T2 and gametogenetin binding protein 2 is associated with drug resistance in ovarian cancer. Oncol Rep (2014) 32:362–72. doi: 10.3892/or.2014.3175 24842157

[26] Świerczewska M Sterzyńska K Wojtowicz K Kaźmierczak D Iżycki D Nowicki M. PTPRK expression is downregulated in drug resistant ovarian cancer cell lines, and especially in ALDH1A1 positive CSCs-like populations. Int J Mol Sci (2019) 20:2053. doi: 10.3390/ijms20082053 31027318 PMC6515253

[27] Borucka J Sterzyńska K Kaźmierczak D Świerczewska M Nowacka M Wojtowicz K. The significance of interferon gamma inducible protein 16 (IFI16) expression in drug resistant ovarian cancer cell lines. BioMed Pharmacother (2022) 150:113036. doi: 10.1016/j.biopha.2022.113036 35489285

[28] Wojtowicz K Sterzyńska K Świerczewska M Nowicki M Zabel M Januchowski R. Piperine targets different drug resistance mechanisms in human ovarian cancer cell lines leading to increased sensitivity to cytotoxic drugs. Int J Mol Sci (2021) 22:4243. doi: 10.3390/ijms22084243 33921897 PMC8073496

[29] McGuire WP Hoskins WJ Brady MF Kucera PR Partridge EE Look KY. Cyclophosphamide and cisplatin compared with paclitaxel and cisplatin in patients with stage III and stage IV ovarian cancer. N Engl J Med (1996) 334:1–6. doi: 10.1056/NEJM199601043340101 7494563

[30] Liu X Gao Y Lu Y Zhang J Li L Yin F. Oncogenes associated with drug resistance in ovarian cancer. J Cancer Res Clin Oncol (2015) 141:381–95. doi: 10.1007/s00432-014-1765-5 PMC11824102 24997551

[31] Januchowski R Wojtowicz K Sujka-Kordowska P Andrzejewska M Zabel M. MDR gene expression analysis of six drug-resistant ovarian cancer cell lines. BioMed Res Int (2013) 2013:241763. doi: 10.1155/2013/241763 23484165 PMC3591129

[32] Watson ZL Yamamoto TM McMellen A Kim H Hughes CJ Wheeler LJ. Histone methyltransferases EHMT1 and EHMT2 (GLP/G9A) maintain PARP inhibitor resistance in high-grade serous ovarian carcinoma. Clin Epigenet (2019) 11:165. doi: 10.1186/s13148-019-0758-2 PMC6882350 31775874

[33] Kondrashova O Nguyen M Shield-Artin K Tinker AV Teng NNH Harrell MI. Secondary somatic mutations restoring RAD51C and RAD51D associated with acquired resistance to the PARP inhibitor rucaparib in high-grade ovarian carcinoma. Cancer Discovery (2017) 7:984–98. doi: 10.1158/2159-8290.CD-17-0419 PMC5612362 28588062

[34] Moore K Colombo N Scambia G Kim B-G Oaknin A Friedlander M. Maintenance olaparib in patients with newly diagnosed advanced ovarian cancer. N Engl J Med (2018) 379:2495–505. doi: 10.1056/NEJMoa1810858 30345884

[35] González-Martín A Pothuri B Vergote I DePont Christensen R Graybill W Mirza MR. Niraparib in patients with newly diagnosed advanced ovarian cancer. N Engl J Med (2019) 381:2391–402. doi: 10.1056/NEJMoa1910962 31562799

[36] . Available at: https://www.ema.europa.eu/en/documents/smop/chmp-post-authorisation-summary-positive-opinion-zejula-ii-19_en.pdf.

[37] Kyo S Kanno K Takakura M Yamashita H Ishikawa M Ishibashi T. Clinical landscape of PARP inhibitors in ovarian cancer: molecular mechanisms and clues to overcome resistance. Cancers (2022) 14:2504. doi: 10.3390/cancers14102504 35626108 PMC9139943

[38] Miller RE El-Shakankery KH Lee J-Y. PARP inhibitors in ovarian cancer: overcoming resistance with combination strategies. J Gynecol Oncol (2022) 33:e44. doi: 10.3802/jgo.2022.33.e44 35320891 PMC9024188

[39] Boussios S Abson C Moschetta M Rassy E Karathanasi A Bhat T. Poly (ADP-ribose) polymerase inhibitors: talazoparib in ovarian cancer and beyond. Drugs R D (2020) 20:55–73. doi: 10.1007/s40268-020-00301-8 32215876 PMC7221042

[40] Bartoletti M Cecere SC Musacchio L Sorio R Puglisi F Pignata S. Recurrent ovarian cancer in the era of poly-ADP ribose polymerase inhibitors: time to re-assess established clinical practices. ESMO Open (2021) 6:100135. doi: 10.1016/j.esmoop.2021.100135 33930658 PMC8100608

[41] Sorio R Roemer-Becuwe C Hilpert F Gibbs E García Y Kaern J. Safety and efficacy of single-agent bevacizumab-containing therapy in elderly patients with platinum-resistant recurrent ovarian cancer: subgroup analysis of the randomised phase III AURELIA trial. Gynecol Oncol (2017) 144:65–71. doi: 10.1016/j.ygyno.2016.11.006 27871723

[42] Bell D Berchuck A Birrer M Chien J Cramer DW Dao F. Integrated genomic analyses of ovarian carcinoma. Nature (2011) 474:609–15. doi: 10.1038/nature10166 PMC3163504 21720365

[43] Agarwal R Kaye SB. Ovarian cancer: strategies for overcoming resistance to chemotherapy. Nat Rev Cancer (2003) 3:502–16. doi: 10.1038/nrc1123 12835670

[44] Galluzzi L Senovilla L Vitale I Michels J Martins I Kepp O. Molecular mechanisms of cisplatin resistance. Oncogene (2012) 31:1869–83. doi: 10.1038/onc.2011.384 21892204

