# Corrigendum: Bibliometric and visualized analysis of drug resistance in ovarian cancer from 2013 to 2022

**DOI:** 10.3389/fonc.2023.1228879

**Published:** 2023-05-31

**Authors:** Jiahua Liu, Junnan Ma, Jiarong Zhang, Chengming Li, Bowen Yu, Hyok Chol Choe, Kaiyue Ding, Liu Zhang, Lin Zhang

**Affiliations:** Institute (College) of Integrative Medicine, Dalian Medical University, Dalian, China

**Keywords:** drug resistance, ovarian cancer, bibliometric, Citespace, Vosviewer, PARP inhibitors, bevacizumab

In the published article, there was an error in the Funding statement. In the Funding statement, we left out a funding. The original Funding statement is “This work was supported by grants from the National Natural Science Foundation of China (No. 81873195), the Liaoning Revitalization Talents Program (XLYC1907113), the Natural Science Foundation of Liaoning Province (2023010109-JH2/1013), and the Distinguished Young Scholars in Dalian (2022RJ19).”. The correct Funding statement appears below.

**Funding**

This work was supported by grants from the National Natural Science Foundation of China (No.81873195), the Liaoning Revitalization Talents Program (XLYC1907113), the Natural Science Foundation of Liaoning Province (2023010109-JH2/1013), the Distinguished Young Scholars in Dalian (2022RJ19), and the Dalian Medical University Foundation for Teaching Reform Project of Undergraduate Innovative Talents Training (111906010210).

The authors apologize for this error and state that this does not change the scientific conclusions of the article in any way. The original article has been updated.

